# A Stakeholder-Centered mHealth Implementation Inquiry Within the Digital Health Innovation Ecosystem in South Africa: MomConnect as a Demonstration Case

**DOI:** 10.2196/18188

**Published:** 2022-06-16

**Authors:** Idon-Nkhenso Sibuyi, Retha de la Harpe, Peter Nyasulu

**Affiliations:** 1 Faculty of Informatics and Design Cape Peninsula University of Technology Cape Town South Africa; 2 Division of Epidemiology & Biostatistics Faculty of Medicine & Health Sciences Stellenbosch University Cape Town South Africa

**Keywords:** MomConnect, mHealth, patient-facing eHealth, digital health innovation ecosystem, practitioner-researcher, stakeholder-centered design, re-engineering in health services, sustainable development goals, principles of digital development, global digital health index, strong structuration theory

## Abstract

**Background:**

The internet is a useful web-based multimedia platform for accessing and disseminating information unconstrained by time, distance, and place. To the health care sector’s benefit, the advent and proliferation of mobile devices have provided an opportunity for interventions that combine asynchronous technology-aided health services to improve the lives of the less privileged and marginalized people and their communities, particularly in developing societies.

**Objective:**

This study aimed to report on the perspectives of the different stakeholders involved in the study and to review an existing government mobile health (mHealth) program. It forms part of a study to design a re-engineered strategy based on the best demonstrated practices (considerations and methods) and learned experiences from the perspectives of multiple stakeholders within the digital health innovation ecosystem in South Africa.

**Methods:**

This study used an ethnographic approach involving document review, stakeholder mapping, semistructured individual interviews, focus group discussions, and participant observations to explore, describe, and analyze the perspectives of its heterogeneous participant categories representing purposively sampled but different constituencies.

**Results:**

Overall, 80 participants were involved in the study, in addition to the 6 meetings the researcher attended with members of a government-appointed task team. In addition, 46 archived records and reports were consulted and reviewed as part of gathering data relating to the government’s MomConnect project. Among the consulted stakeholders, there was general consensus that the existing government-sponsored MomConnect program should be implemented beyond mere piloting, to *as best as possible* capacity within the available resources and time. It was further intimated that the scalability and sustainability of mHealth services as part of an innovative digital health ecosystem was hamstrung by challenges that included stakeholder mismanagement, impact assessment inadequacies, management of data, lack of effective leadership and political support, inappropriate technology choices, eHealth and mHealth funding, integration of mHealth to existing health programs in tandem with Goal 3 of the Sustainable Development Goals, integration of lessons learned from other mHealth initiatives to avoid resource wastage and duplication of efforts, proactive evaluation of both mHealth and eHealth strategies, and change management and developing human resources for eHealth.

**Conclusions:**

This study has only laid a foundation for the re-engineering of mHealth services within the digital health innovation ecosystem. This study articulated the need for stakeholder collaboration, such as continuous engagement among academics, technologists, and mHealth fieldwork professionals. Such compelling collaboration is accentuated more by the South African realities of the best practices in the fieldwork, which may not necessarily be documented in peer-reviewed or systematic research documents from which South African professionals, research experts, and practitioners could learn. Further research is needed for the retrospective analysis of mHealth initiatives and forecasting of the sustainability of current and future mHealth initiatives in South Africa.

## Introduction

### Background

The term *digital health innovation ecosystem* (DHIE) entails the systematization of digital health networks, environments, and communities (stakeholders) relying on information and communication technology (ICT) to connect and relate with each other for the purpose of improving health services. Furthermore, it empowers patients to manage their well-being and that of their families and communities [1]. The process of developing and designing a health service requires an in-depth understanding and knowledge of the ecosystem, as well as holistic consideration from different stakeholder perspectives [2]. Accordingly, the design process is intended for the use and distribution of a (health) product at any moment and at any of its location points to people during the product’s lifetime, to the advantage of those whose needs are not appropriately addressed. In the context of this study, the centralization of the DHIE underpins the researcher’s effort to explore and identify the fundamental tenets in the design, application, and implementation of a digitalized health product with respect to its entire value chain of networks, environments, and communities [3]. The networks in the ICT-dominated ecosystem or environment are constituted by health care stakeholders, institutions, and devices. Such an environment is characterized principally by the successful implementation of interactive digital best demonstrated practices and solutions [4]. In this study, the DHIE is pivotal, as it is the term or concept or phenomenon around which the implementation efficacy (or otherwise) of the MomConnect project (as a demonstration case) could be determined as an initiative of the National Department of Health (NDOH) in its efforts to introduce and implement a national rollout of the digital health strategy for South Africa [5].

Given the aforementioned context of the DHIE, this study focuses on an important topic pertaining to the implementation of the MomConnect project, which has had a nationwide rollout in South Africa. It is not a small-scale pilot project limited to several facilities or a single district but has been implemented nationwide and emphatically referred to since 2014 by previous Ministers of Health and is currently in their annual health budget speeches as an indication of the government’s irreversible trajectory toward the digitization of health services as a means to broaden access [5,6]. Therefore, this research on sustainability issues and the factors affecting its success is critical.

Technology-assisted health care service delivery is regarded as the new frontier of innovations in health care, facilitated by improvements in ICTs and the internet’s asynchronous interconnectivity [4]. Mobile health (mHealth) technology—part of eHealth—is a multidisciplinary field cutting across health care, medical, and technological sciences, connecting medical informatics, business, and public health through internet-based technologies [7]. At the same time, experience has shown that the scalability and sustainability of mHealth services as part of an innovative digital health ecosystem could be hamstrung by factors such as stakeholder mismanagement, lack of political support, appropriate choice of technology, funding, and integration of mHealth into existing health programs in tandem with the Sustainable Development Goals [7].

In many developing countries, innovative initiatives aimed at enhancing the delivery of health services and disease management have been stifled by *pilotitis*—defined as a state of perpetual preliminary testing of projects in terms of which many technological innovations and initiatives, including mHealth, have not progressed to their intended full capacity [8]. A perennial state of *pilotitis* is caused by, among other factors, inadequate monitoring and evaluation systems, weak interorganizational and intraorganizational control mechanisms, and poor in-country digital architectures [8]. *Project Kopano* in South Africa and *Hello Mama* in Nigeria are examples of projects that stagnated because of pilotitis and did not develop beyond their pilot phases. Consequent to *pilotitis* and its negative organizational impacts, the objective of addressing Sustainable Development Goal 3 (good health and well-being) is jeopardized, as health technological developments are not accessible to most people for whom health care service provision is an absolute requirement [9].

The digital management of diseases through mHealth and eHealth reflects the development, adoption, and integration of ICT-based innovations in health care service planning, management, and delivery [10]. However, health care facilities and systems in many developing countries are still paper-reliant in many parts of their operations, which stifles progress insofar as digitally improving the quality, safety, and productivity of their health care services is concerned. mHealth has ushered in important changes through its facilitation of access and willingness to use portable devices for health care needs. mHealth is a medical and public health system that promotes the enhancement of health services with the support of wireless multimedia technologies such as mobile phones and other devices for monitoring patients and their recovery progress and well-being [11]. mHealth and eHealth have the potential to support and strengthen existing health care programs, rather than focusing on the discovery of new treatments and the development of clinical interventions by themselves [12]. Such a complementary approach in developing countries is significantly helpful, considering the plethora of factors such as adverse conditions (eg, poverty), limited investments, and resource constraints (eg, provision of safe drinking water, sanitation, basic education, medicines, and skilled personnel). For example, patients’ adherence to medication could be enhanced through mHealth, rather than administering new medication when the only issue was adherence (a factor of educating patients), and not a reflection on the effectiveness of the health care system in general or a particular program within the health system.

The application of mobile technology–based health care services (mHealth) is a credit associated with the introduction of ICT in health development. However, the design strategies and capabilities of such devices have also been questioned [11]. A differentiation between *user-centered* and *stakeholder-centered* designs is worth mentioning. In information technology systems architecture or design science, the 2 concepts are used interchangeably, although *stakeholder-centered design* is more applicable in the context of public health systems. In this regard, a stakeholder-centered design perspective was adopted in this study. A stakeholder design is defined as a process intended for the use of a (health) product at any moment and at any of its location points by all people during the product’s lifetime to the advantage of those whose needs are not appropriately addressed [13]. Every effort should be made to identify and understand these stakeholders and address their needs to keep the chain intact [13].

The aim of this study was to share the perspectives of the different stakeholder groups involved in the design and implementation of a mobile app used in maternal health care services in a particular DHIE. The research question that guided the research for this paper is: What are the perspectives of the different stakeholder groups in the form of best practices, lessons learned, or issues identified based on their involvement in the design, development, and use of a specific mHealth application? The insights gained from this study should assist in moving health innovations beyond the pilot stage.

### Background to the MomConnect Project of the NDOH

The MomConnect project is a product of the NDOH’s National Digital Health Strategy (NDHS) intended to meet health targets and simultaneously maintain the momentum thereof, that is, scalability and sustainability [2,5]. The NDHS itself (launched in 2019) incorporated and combined eHealth and mHealth strategies, both of which have been periodically reviewed every 5 years since 2014 (the year of MomConnect’s official launch). Conceptually, eHealth premises on the provision of health care through ICT to empower patients, families, and communities in the improvement, monitoring, and management of their health and well-being [11]. Meanwhile, mHealth relates to medical and public health practices and disease management programs supported by a range of interactive ICTs such as mobile phones, patient monitoring devices, and PDAs [1,14]. In essence, the current NDHS (2019-2024) is cognate from the review of the National eHealth Strategy (2012-2016), which focused mainly on improving governance systems and structures, integration of information systems, and technological enhancement and interface of the health care system and its users [6].

The merging of eHealth and mHealth by the NDOH signifies the need for critical considerations for a re-engineered and coordinated mHealth strategy that incorporates funding for eHealth and mHealth, impact assessment, management of data, effective leadership and governance from the NDOH, integration of lessons learned from other mHealth initiatives to avoid resource wastage and duplication of efforts, proactive evaluation of both mHealth and eHealth strategies, and change management and developing human resources for eHealth [5,6]. Moreover, contemporary issues of mHealth services could be addressed by applying digital development principles to strengthen best practices and address existing implementation gaps [10]. Furthermore, the centralization of a digital health system at the NDOH was viewed as a critical step in ensuring coordinated governance and effective leadership [6].

As espoused by the NDOH, the vision of the NDHS (2019-2024) is premised on the betterment of all South Africans’ lives through digitized person-centered health services within an ecosystem inhabited by people and technology-driven processes [6]. The prioritized outcomes for better health for all South Africans rested on the key strategic pillars of person-centeredness, broadening access to health services, innovative sustainability, workforce-inspired economic development, and an interdepartmentally collaborative government approach. The strategic components of the digital health strategy are also mention worthy, as they cohere with some specific reference the study has made of the NDHS-MomConnect linkage, most of which have also emerged as critical variables in the findings. These 9 strategic components in the NDHS documents are leadership, stakeholder engagement, investment in strategy, governance, systems architecture and standards, digital apps and their services, connectivity infrastructure, legislation, policy and compliance, and workforce capacity (for economic development).

### An Overview of MomConnect

The MomConnect project (which is not the strategy per se and is the demonstration case and overarching point of reference in this study) was officially launched in August 2014 as the NDOH’s initiative for the improvement of maternal, child, and women’s health (MCWH) services [6]. This project was founded on key elements, all of which are interstitially associated with maternal and child health and well-being until the newborn child is aged 1 year [5,6]. These elements are subscription (of users and beneficiaries to the mobile service), registration (of users on the national database through a common Unstructured Supplementary Service Data [USSD] number with the assistance of health care workers at the nearest health facility), SMS text messaging (NDOH SMS text messages relating to all relevant MCWH information), service rating (use of the free USSD number to rate the service using mobile devices), and compliments and complaints by users to local districts.

From its inception, this project was viewed as the *flagship* or standard of digitally propelled national programs of care for maternal and child health services in health care facilities across South Africa (Pillay Y, BP, unpublished data, April 2022) [5]. MomConnect was proudly welcomed by virtually all its beneficiaries, namely, the health care users who were mothers using MCWH services, pregnant women who came for antenatal care (ANC), and mothers who came for postnatal care. Notwithstanding some challenges, some observable success factors of MomConnect (since August 2014) included registration of >1.9 million users, a minimum daily rate of 1000 SMS questions asked by users, 9 times more compliments received than complaints, and additional specific elimination of mother-to-child transmission messaging for women who have been HIV positive since September 2016 [6]. Nurses and auxiliary personnel at public health care facilities encourage and help pregnant women and new mothers register for MomConnect. Once registered, these women receive 2 to 3 SMS text messages per week based on their stage of pregnancy and through the child’s first year of life. MomConnect message content is timed to the expected month of delivery and covers topics such as vaccination and checkup reminders, exclusive breastfeeding recommendations, psychosocial parenting tips, and baby development.

Given MomConnect’s envisaged national impetus since its inception, it is therefore fait accompli that its scalability and sustainability factors would constitute the most salient variables within the DHIE parameters. The scalability and sustainability of a large-scale project such as MomConnect are achieved optimally by meeting targets and maintaining the momentum at the same time [2]. In general, project scalability and sustainability investigations are necessitated by challenges that were likely to relegate such potentially viable eHealth and mHealth services to a state of *pilotitis* [15]. Scalability most profoundly relates to quantitatively broadening or increasing access to health care services, whereas sustainability is concerned with qualitatively ensuring long-term success and continuation with unconstrained availability of financial, human, and infrastructural resources [5,8]. However, the current environment of MomConnect’s implementation is still characterized by environmental factors reminiscent of a state of *pilotitis*. For instance, the implementation task team was focused more on scale (digital services expansion) than on sustainability (long-term durability) from the beginning of the initiative. It was performance driven, based on achieving targets. Once there was sufficient (quantitative) achievement on the target side, sustainability (qualitative) became a casualty. In addition, critical factors that were moderate at MomConnect’s inception in 2014 became more critical, such as fundraising, exploring the registration of MomConnect as an independent entity, equitable allocation of contracts to competent digital service providers, stakeholder conflicts of interest when appointed to the board of the new entity, and perpetual legal advice being sought, among others.

The original MomConnect content was created by BabyCentre, adapted for the South African context by a team of local experts, customized for the length of an SMS text message (160 characters), and reviewed by a panel of experts including maternal health clinicians. A component of MomConnect has a task team that meets the most active stakeholders monthly [16]. MomConnect was selected as a case example in this study because it is the first nationally scaled-up mHealth service. An in-depth study of its implementation from a service design perspective may assist in obtaining evidence-based and best-practice mHealth implementation strategies. From a technical perspective, mHealth implementation also includes user-provider ethical components such as trustworthiness, privacy, and confidentiality.

As South Africa’s first national-scale mHealth service, the implementation of MomConnect has the potential to fill the knowledge gap in mHealth implementation dynamics (which may include service design) [17]. There is currently limited published research in Sub-Saharan Africa on large-scale mHealth implementation on which to establish empirical investigation pertaining to the exploration of the well-designed efficacy and effectiveness of mHealth services [17]. The latter view is supported by the fact that the proliferation of mHealth initiatives in many developing countries has not necessarily translated into rollouts at the national level, as well as the practical implications of these projects on the routines of the facilities at which they have been rolled out [18] (NDOH, 2014).

### Study or Research Framework

The study framework shown in Table 1 depicts 3 critical variables, namely, various categories of participants as the sources of data, research methods or instruments through which particular types or forms of data were generated, and the approach adopted to analyze the accumulated data. Necessarily so, these 3 diagrammatically represented variables are also indicative of the *route* of the study, that is, the most salient processes and activities performed throughout the study from its inception and conclusion [19,20]. The approach adopted by the researcher in analyzing the accumulated data in this predominantly qualitative-ethnographic study is critical and mention worthy, because it (approach) underpins the logic and rationale for the study and all its associated processes, which are captured to varying degrees of detail, particularly in the *Methods* and *Results* sections.

Regarding the aforementioned approach, the study framework reflects and encompasses a practitioner-researcher perspective in terms of which the researcher is simultaneously an active participant or observer in the situation being analyzed [21]. In addition, the practitioner-researcher approach has been influenced by the fact that the researcher is also a public health practitioner with >10 years of experience in the field. Moreover, the researcher served as a member of the MomConnect Task Team from November 2015 to June 2018 when he was employed by one of the implementing partner organizations and seconded to the NDOH to implement the elimination of mother-to-child transmission of the HIV component of the MomConnect initiative. This was the period during which the various critical activities and processes of the study were conducted.

Largely as a factor of the practitioner-researcher approach, the research approach depicted in Table 1 highlights the practitioner-researcher perspective as influential in shaping the study framework with respect to the chronology of the research process as clearly demarcated in the 2-phased stages (January 2017 to June 2018) showing the empirical and nonempirical (eg, document review) domains of the investigation. It is also clear from Table 1 that all the research-related activities and processes are cohesively bound and characterized by 2 indispensable components: simultaneity and contiguity.

**Table 1:** Depiction of study framework.

Stages of research and participant category and source of data			Activity period		Research method and type of data		Mode of data analysis and analytic tool
**Phase 1**							
	MomConnect repository and archived data (including minutes and reports dated February 2014 to June 2018)	January 2017 to June 2018		Document and literature review		Content analysis	
**Phase 2**							
	Ministerial Advisory Committee on eHealth	January to December 2018		Interviews		Thematic analysis	
	**MomConnect task team**						
		January to December 2018		Stakeholder relationship mapping		Discourse and conversational analysis	
		January to December 2018		Interviews		Thematic analysis	
	MomConnect task team meetings	January to June 2018		Ethnographic observations		Discourse and conversational analysis	
	Clinical staff	January to December 2018		Interviews		Thematic analysis	
	Auxiliary health personnel	January to December 2018		Interviews		Thematic analysis	
	Patients or health service users	January to June 2018		Focus group discussions		Thematic analysis	

The notion of contiguity entails the inseparability of all relevant research variables, whereas simultaneity encompasses the development, occurrence, or undertaking of more than a single activity or process simultaneously [19,20]. For instance, all phase 2 processes and activities are contiguous with the core phase 1 activity. Inversely, the review of pertinent literature and relevant documents occurred throughout the research process. Thus, these 2 phases are contiguous on the basis of their complementarity as well. By contrast, all phase 2 activities and processes happened at different times and places in real time yet simultaneously in the context of the broader period during which they occurred and were performed.

Table 1 also clearly illustrates a multimodal approach of data collection and analysis, which justifies reference to convergent thinking and analysis as a tool for understanding the final outcomes and consequent findings, in terms of which the broader domain of MomConnect’s implementation strategies could be justifiably assessed [20,22].

## Methods

### Research Setting

The study was conducted at 2 geographically disparate locations in the Gauteng Province, South Africa. The first setting was the most appropriate, given that it was the physical location for the offices of the NDOH, the fiduciary custodian of the MomConnect project. In addition, members of both the MomConnect Task Team and the Ministerial Advisory Committee on eHealth (MACeH) were more accessible at this site, as they regularly attended their meetings at the NDOH’s offices. This venue was also crucial because it housed the archived documents in the MomConnect repository. These documents were instrumental in secondary data collection.

The second setting consisted of 4 inner-city primary health care (PHC) facilities (clinics) located in the largest city and economic hub of the country. These 4 clinics were selected because they were located in high-density populations, which ensured large-scale involvement of the selected participants. Furthermore, each of the 4 study clinics in Johannesburg’s Region F offers similar PHC services that predominantly focus on HIV or AIDS.

### Participant Characteristics and Their Recruitment

A total of 5 stakeholder categories were involved in this study, each representing both national-and facility-level perspectives. The first category, the MACeH and health facilities, was appointed by the Ministry of Health and is responsible for advising the Minister of Health on policy-related matters concerning eHealth. The second category, the MomConnect Task Team, involved representatives of different private and public sector organizations, academic institutions, and independent consultants. These representatives were involved at different stages of the MomConnect implementation and have held monthly meetings since the inception of the MomConnect project. The third category involved professional clinical staff at the 4 inner-city PHC facilities. Fourth, there were also auxiliary health personnel based at the same health care facilities. The fifth and final informant category consisted of patients or health care users. Table 2 illustrates the 5 stakeholder categories, as well as the primary data collection methods used for each.

**Table 2:** Participant categories sampled and their data collection methods (N=80).

Participants and stakeholders	Composition	Count, n (%)	Site and data collection method
Ministerial Advisory Committee on eHealth	Consists of 1 senior government official representative from each of the 9 provinces, academia, research organizations (eg, CSIR^a^), and private sectors, as per the government gazette	9 (11)	1-on-1 (face-to-face, telephonic, and virtual) interviews at NDOH^b^ offices
MomConnect Task Team	NDOH officials and implementing partners (academia, funders, NGOs,^c^ consultants, and research institutes)	15 (19)	1-on-1 (face-to-face, telephonic, and Skype) interviews at NDOH offices
Clinical staff	Professional nurses working at the 4inner-city clinics providing ANC^d^ services in Johannesburg Region F	5 (6)	Face-to-face interviews at the inner-city PHCs^e^
Auxiliary health personnel	Staff based at the facility who are not registered clinicians but do interact with patients who come for ANC services, for example, health promoters, lay counselors, community health workers, and data capturers within the health care facility	6 (8)	Face-to-face interviews at the inner-city PHCs
Patients or users	Pregnant women and mothers visiting health care facilities for maternal, child, and women’s health at the clinics; women who were at the facilities on the specific day when researchers were at the clinic were all sampled and formed part of FGDs^f^	45 (56)	Five focus group discussions of nine members each, at the inner-city PHCs

^a^CSIR: Council for Scientific and Industrial Research.

^b^NDOH: National Department of Health.

^c^NGO: nongovernmental organization.

^d^ANC: antenatal care.

^e^PHC: primary health care.

^f^FGD: focus group discussion.

### Sampling of Research Sites and Participants

Purposive sampling was used for the selection of all key informants, based on our own professional judgment, experience, and knowledge of the research environment, informing us that the sampled participants complied with the requirements or criteria that we determined before the execution of the empirical data collection phase [20,23]. Nonprobability convenience sampling was used for the selection of the 4 inner-city research sites based on their ready availability and easy accessibility [9]. Both sampling methods were motivated by the fact that the researcher was a member of the MomConnect Task Team from October 2015 to June 2018. In this capacity, he was appointed as a digital health expert and seconded to the NDOH.

### Description of the Different Research Methods

The MACeH representatives were selected mainly for policy-related reasons, given their knowledgeability and close interactions with the Ministry of Health. In contrast, the MomConnect Task Team members were sampled based on the fact that they represented different stakeholder constituencies involved in the implementation of the MomConnect project at the national, provincial, district, and subdistrict levels; attended the MomConnect Task Team meetings regularly; and were very knowledgeable about the project’s functioning, mandate, and expected deliverables when appointed by the government. This team consisted of representatives of different private and public sector organizations, academic institutions, and independent consultants who have been involved at the different stages of the MomConnect project’s implementation since its inception in 2014. Professional clinical staff were purposively selected based on their practice-related knowledge and work experience pertaining to the functioning of their inner-city health care facilities in Johannesburg. Meanwhile, the selection of auxiliary health personnel professional staff was influenced by our concern with perceptions of their exclusion in the MomConnect activities, although they were expected to render services related to the requirements of MomConnect users.

In contrast, the category of the purposively sampled health care service users were mothers who using MCWH services, pregnant women receiving ANC, and mothers visiting the facilities for postnatal care. These were the most direct beneficiaries of the government’s MomConnect initiative, and their knowledge, experiences, and perceptions were indispensable to this study.

### Data Collection Methods and Processes

#### Overview

In essence, this study was conducted in 2 phases that are distinguishable by their contiguity and simultaneity elements. In varying degrees, both Tables 1 and 2 provide significant information that also preludes the trajectory or approaches adopted for the data collection and analysis processes. It is worth noting that by virtue of the study’s broader domain of simultaneity and contiguity, the data collection and its associated analysis processes have seamlessly (rather than chronologically) integrated a methods-based orientation for data collection and a participant-based orientation for the framework of results. Whereas the methods-based orientation inevitably emphasizes and accentuates the data, the participant-based orientation prioritizes and particularizes the source of the data itself. As such, relevant data were collected according to the research methods described below.

#### Literature and Document Review

The MomConnect repository itself was relevant, as it housed the official institutional memory of the MomConnect Task Team. This phase of data collection was of significant importance to the study, as it facilitated the evaluation of MomConnect’s decision-making processes, providing a comprehensive background on the implementation process of the project, as well as an opportunity to examine the difference between the planned and actual implementation of the MomConnect project.

The document review included the official government’s (NDOH’s) MomConnect initiative work plan and other records. In this regard, the MomConnect repository, located in the NDOH, was also a reservoir of information, including the minutes of the MomConnect Task Team meetings. The MomConnect repository contains publicly available documents such as progress reports of implementation, archived and current minutes of task team project meetings, survey reports, and data and operational research documents or reports.

#### Stakeholder Relationship Mapping

Stakeholder relationship mapping basically refers to the identification and categorization of the main project participants (individuals, organizations, or institutions) who directly or indirectly have a vested interest in the ultimate outcome of the particular project based on their levels or stages of involvement in the very same project [24]. Stakeholder relationship mapping was of critical importance, especially because poor and weak program management challenges accounted for the failure of scalability and sustainability capacity required for the delivery of huge national projects such as MomConnect [25,26]. Therefore, stakeholder mapping was only applied to the participant category located within the decision-making and policy development echelons (such as the members of the MomConnect Task Team) rather than to the project implementers (eg, clinical and auxiliary health personnel) or end users (ie, patients at the PHC facilities). In this study, relationship mapping was only applied to the MomConnect Task Team members through a written exercise, filling-in an informed consent form, and returning to the researcher on the same day. After informed consent was obtained, task team members were emailed an exercise used to determine the nature and range of their past and current relationships that may have some impact on the MomConnect project.

Having obtained considerable background information and knowledge through the systematic review of relevant literature and documents, stakeholder mapping (similar to research participant selection criteria) constituted the logical phase before the actual empirical data collection itself through interviews and focus group discussions [27]. This mapping of the various groups and stakeholders was critical as it enhances more understanding of the relationships and interrelatedness of individuals, groups, and the specific mHealth service itself. The mapping process is focused on the exploration and understanding stakeholder relationships rather than on finding out *who* they are [28].

#### Semistructured Face-to-face Interviews

This phase of data collection was conducted with the MomConnect Task Team members, MACeH, and clinical and auxiliary health personnel.

#### Ethnographic Observation of MomConnect Task Team Meetings

In research involving an empirical component, the observation of participants is an ongoing process (Pillay Y, BP, unpublished data, April 2022). From an ethnographic perspective, the observation of research participants further provides an opportunity for the researcher to interact directly in conversations or dialogues with the participants and observe their attitudes, behavior, and interaction toward each other and one another within their ecological parameters of the natural environment to which they are very familiar and in which they interpreted their reality and conditions [29,30]. In this regard, participant observation complemented both the primary data collection methods (ie, individual interviews and focus group discussions).

Participant observation was facilitated by means of the researcher’s physical observation of and listening to MomConnect Task Team members in their monthly meetings. The observation rationale was to add value to this study to the extent that more understanding was important for assessing the stakeholder relationships and collaboration. The latter 2 aspects were critical factors because they provided a basis for the relative determination of the success or failure of project design, planning, and implementation processes and dynamics at both the bureaucratic and technocratic levels, given the composition of both the MACeH and the MomConnect Task Team [19,31]. For the purpose of this study, and given the heterogeneous representation of interests and constituencies within the MomConnect Task Team, 3 of their monthly meetings were attended. Field notes were taken, focusing on their interpersonal relationships, as well as their decision-making processes and procedures in meetings. This phase was complementary to the review of relevant MomConnect repository documents.

#### Focus Group Discussion With Service Users at Inner-City PHC Facilities

As shown in Table 1, this targeted form of engagement, interaction, and conversations was conducted with pregnant women who came for MCWH services and ANC and mothers who came for postnatal care. Focus group discussions were advantageous in that virtually all participants were more at ease sharing and learning in a group from the experiences, knowledge, and perceptions of others [32].

### Data Analysis Approaches

Both the heterogeneous nature of participant categories and the range of ethnographically oriented instruments of data collection triangulated the data analysis approach involving content, thematic, discourse, and conversational analysis. The study emphasizes that, despite their terminological variation, the modes of analysis all fundamentally focus on deriving meaning from themes developed from the content of empirical and nonempirical research methods used in this study [18].

### Ethics Approval

Ethics approval was obtained from Cape Peninsula University of Technology. Furthermore, access to conduct research was endorsed by the NDOH, Gauteng Department of Health and City of Johannesburg.

## Results

### Overview

In contradistinction with the methods-based approach of data collection, the results framework presented in Table 3 is emblematic of a participant-based orientation. As such, the results or findings are presented based on the type or category of participants as providers or sources of the self-same data obtained through different methods. Notwithstanding the transcendence of participant categories over the different data collection methods in this regard, both methods and participants are still contiguously linked. There could be no empirically generated data without participants as the vital source of the information sought to fulfill the study’s aim [1,20]. Accordingly, the results framework in Table 3 is the outcome of the participant-method contiguity. Most importantly, the results reflect the convergence of data analysis methods, that is, the predominant thematic mode complemented by conversational and discourse analysis.

Owing to the variability of research methods applied and the vastness of the data collected, the results are presented in varying degrees of detail consonant with aspects they address in relation to the development, implementation, and usability of the MomConnect system of digitizing health care services *for all South Africans* [6]. From the perspective of this study, the development-implementation-usability continuum is pivotal in determining the extent to which the NDOH’s MomConnect initiative could be demonstrated as a case of policy or strategy success or failure with respect to its attendant sustainability and scalability factors.

Table 3 is reflective of the eventual outcomes of various data analysis processes adopted (Table 1) to construct meaning from various participant categories representing various components and aspects in the MomConnect policy development, strategy design, and implementation and health care service users’ benefit. In the end, the convergence of the various data analytic modes also reflects the inextricability of participants’ environmental dynamics (eg, vested interests and influences) and the inevitable researcher-practitioner approach adopted. Owing to the vastness of the data collected, the emergent themes have been paired globally in groups rather than individually.

**Table 3:** Results framework.

Participants and main theme			Main category	Subcategory
**Ministerial Advisory Committee on eHealth (interviews)**				
	Governance and leadership		mHealth^a^ centralization	mHealth service rationalization
	Strategy integration		eHealth and mHealth strategy perceptions	Strategy application feedback
	Stakeholder involvement		Clinicians, technophobia or capacity building; mHealth providers and consumer engagement	—^b^
	Research and development		Demonstrable evidence of implementation outcomes or impact; mHealth piloting perspectives	—
	**Service continuity**			
		Sustainability		Total cost of ownership and co-utility; cost of ownership and cost utility; outsourcing culture
		Scalability		NDOH^c^ financial and human resources; provincial realities regarded as mHealth barriers; computing infrastructural issues
		Design thinking		Lessons learned
		Service implementation		Human and financial resources
	**Ecosystem or environment**			
		Organizational		Vision, policies, and guidelines; governance and leadership; political authority or oversight
		Ethical aspects		Privacy and security; data ownership
		Integration		Technical: infrastructure and interoperability
**MomConnect Task Team (minutes)**				
	**Service conceptualization**			
		Stakeholder considerations		Facility-level consultation and collaboration
		Design process		Considerations; research and expansion considerations
		Integration		Technical: infrastructure and interoperability
	Roll out		National to provincial scaling-up process; operations and performance	—
	Service continuity		Service continuity; sustainability and evolution	—
**MomConnect task team (interviews)**				
	**MomConnect as a case example**			
			Member relationship mapping	—
			Support for MomConnect as a case example; differing piloting views	—
	Critical considerations		mHealth and eHealth strategy	Life span within NDOH or integration of initiative within health programing; ethical service implementation; uncertainty over sustainability
	Ministry of Health prerogatives		Leadership and management; teamwork; operations; recommendations	User-centered design; sustainability; change management; stakeholder management
**Facility-level (clinicians, auxiliaries, and service users)**				
	**Service touch point capacity**			
			Stakeholders	Involvement of nurses or clinicians (capacity building and NurseConnect); mothers, pregnant women, and caregivers; foreign nationals (challenges)
			Service implementation	Content of information; ethical considerations; MomConnect helpdesk and interactive communication with nurses; subscription and marketing; service rating or feedback
			Health care facility environment	—
			Operations	—

^a^mHealth: mobile health.

^b^Not available.

^c^NDOH: National Department of Health.

### Findings Emanating From the MACeH

A total of 6 main thematic statements and their associated categories and subcategories emerged and are discussed in the next sections.

#### Governance and Leadership

Especially in developing countries, governance and leadership issues have been major determinants of the sustainable implementation of health programs and services [25]. In this regard, the MACeH interviews reflected general consensus for the support of centralizing mHealth governance and control in the NDOH: “In itself, the centralization factor entails both the pros and cons of leadership and governance issues.” In this case, centralization was viewed as beneficial because there were a number of unsupervised health initiatives throughout the country, and strong recommendations were mooted for a national database or register to track the performance of mHealth in various parts of the country. Such a step was viewed as advancing the issues of both scalability and sustainability from the very inception of any health care initiative by the Department. This was also viewed as a transparent process that would reduce wastage and duplication of resources. Such a trajectory would ensure that different mHealth implementers are fully acquainted with what other stakeholders are doing. Consequently, mHealth implementers would be capacitated to build on what already exists and learn from the experiences of others. Notwithstanding its advantages, some disadvantages of centralization were also observed. The bureaucratic nature of a centralized system implied that implementers take a long time to obtain permissions, which causes an incremental loss of opportunities during delays [12]. In addition, little or no centralization inadvertently encouraged everyone everywhere to have their own few mHealth projects that never scaled up and remained mostly unknown to the NDOH. In such instances, the need for centralization could be balanced with the need for the devolution of authority, for example, by decentralizing certain implementation functions and regulatory authority to the provinces, regions, and districts [23]. Rationalization relates to the justification of a course of action associated with any aspect of the MomConnect system.

#### Strategy Integration

MomConnect is a product of the overall digital health strategy of the NDOH, and the finding is that the mHealth strategy is not sufficiently integrated into either the eHealth strategy or the health strategies of the country: “I think through that we are missing an opportunity to ensure that the various types of mHealth that is being practiced in the country is fully aligned with the health transformation work that is being led by the ministry and department.” An mHealth strategy needs to be supported by a solid implementation strategy.

#### Stakeholder Involvement

Clinicians were not effectively involved in the system’s design: “...the health system is completely separate from the PHC system.” For instance, the scant involvement of nurses could stereotypically portray them as *technophobic* and stifle their capacitation and competence [3,8].

#### Research and Development

Research on digital health is crucial, considering that mHealth is growing constantly and continuously as part of eHealth and is a component of all aspects of health (prevention, diagnosis, treatment, and research) [7,9]: “*...*at the time MomConnect was rolled out there was not enough substantiated evidence to support national roll out.” It was regarded as a politically driven project: “...we didn’t talk at all around research which I think is an ongoing real problem*.”*

#### Service Continuity

Service continuity is a direct reference to the long-term duration of any program or project [10,13]. In considering the scalability and sustainability factors, the MACeH was also guided by weighing costs associated with sole or co-ownership and outsourcing of certain aspects such as whether funding has been planned for continuing with mHealth projects past the DHIE stage. There needs to be a process of assessing the sustainability of the mHealth initiatives by the relevant stakeholders to ensure that all risks have been evaluated. There is a need for standards, interoperability, and human and financial resources to render the service implementable. Women will be empowered through the MomConnect project, resulting in them having information expectations that could then place a burden on clinics and hospitals to also meet that expectation:

The NDoH uses consultants, that consultant has a life span at the department, you can only employ that person for so long. If you want to be scalable and sustainable you must write a standard approach that is not vendor biased and can be supported by any software vendor.

How can we sustain anything if 50% of it is from donors.

#### Ecosystem or Environment

A digital ecosystem encompasses the entire community of networks within the digital environment to establish best practices [1]. The NDOH was the central authority to safeguard MomConnect users from, among others, protecting their privacy: “Start looking at security issues that may influence the project. [Informed] Consent and POPI [Protection of Personal Information Act of 2013]...” There may be concerns regarding confidence in the system [3]. Scalability and sustainability should guide implementation at the provincial and district levels.

### Findings Emanating From MomConnect Task Team Minutes

The themes are discussed in the next sections.

#### Service Conceptualization

Service conceptualization involves developing and designing a program (at the theoretical or planning stages)—in this case, the MomConnect initiative—such that its technological or digital relevance to and application by the intended users or beneficiaries achieves the required benefits of broadening health care services to all South Africans (Pillay Y, BP, unpublished data, April 2022) [33]. The MomConnect service conceptualization was captured in this study in terms of the affected stakeholders, facility-level consultation and collaboration, and the acceptability (or otherwise) of the design process value chain. During the conceptualization of the service, stakeholders were not only confined to nursing staff: From the afore-cited minutes, it is evident that effective stakeholder considerations extended beyond the nursing personnel as implementers at the facilities. Such a conceptual orientation stood the MomConnect service’s implementation in good stead, as collaboration and cooperation were critical for health care service delivery [34].

#### Design Process

The process of developing and designing a health service requires an in-depth understanding and knowledge of the field, as well as consideration of multidisciplinary perspectives [8,13]:

...research with nurses to improve user-centred design...They [mobile service designers and providers] are also going to do research that will allow us [Task Team/policy makers] to understand which handsets our clients are using...back-end system, mobile operators, service providers, messaging subgroup rep, registration process, communication strategy, launch, field testing...If we design the system properly, we might be able to have them [patients] enter data in the waiting room.

The fact that MomConnect is still in existence in all 9 provinces despite initial imbalances bears testimony to extrinsic factors such as the role played by reputable service designers and providers outside of the NDOH policy-making value chain [32,34].

#### Integration

The willingness to integrate the MomConnect initiative and not implement it as a silo was recorded. However, there were no documented indications of any other health service initiatives to which MomConnect could be integrated. MomConnect was initially conceived as a standalone digitized health service. Only data integration was explicitly mentioned in terms of linking MomConnect data to existing health information system (HIS) data for reporting. The South African ID has been integrated into biometric ID systems that are compliant with various forms of DNA analysis and has been proposed as an identifier for the system. However, foreign national ID numbers must also be captured. The system was also unable to capture dates of birth for underaged pregnant mothers who did not have an ID or foreign nationals who did not have their passports when registering:

To allow for maximum interoperability there should be a number of ID types which will allow all people eligible to receive care to be registered. In South Africa, everyone has a right to emergency medical treatment. Therefore, identifiers which cover foreign nationals, refugees and people without documents should be used.

#### Infrastructure and Interoperability

The technical aspects of mHealth are the quintessential reflection of the integration or interface of ICT systems for human services [15]. The technological infrastructure required for connectivity at a national scale was enhanced by strong NDOH partnerships with established mobile phone operators to boost the economies of scale and size, suggesting that it is technically possible to deliver mHealth interventions to large populations at low cost because downloading and automation to send SMS text messages can be once-off processes [26,35,36].

#### Rollout

The proliferation of mHealth initiatives in many developing countries has not necessarily translated into rollouts at the national level, considering the practical implications of these projects on the daily routines of the facilities at which they have been rolled out [18]. Therefore, the capacity of the NDOH to roll out MomConnect as a national service to its intended beneficiaries was an important determinant of whether this service could be implemented on a large scale [25]. In the context of the MomConnect Task Team minutes, rollout and expansion of the service were both geographical (throughout all 9 provinces) and technological (operations and performance). There were efforts to prevent the MomConnect project from another project that remains in the pilot state:

...*[the Health] minister has announced [on 30 July 2014] that he will be embarking on road shows to introduce MomConnect to the health professional in each province. Demonstration sites were selected, communication materials were approved and translated into other official languages. Establish an ongoing system of formal evaluations and review of potential mHealth projects.*

#### Service Continuity

Determining service continuity was necessary to establish measures recorded in the minutes to ensure the survival of MomConnect beyond its official launch in August 2014. Other than their involvement in providing their network infrastructure for MomConnect use, the role of established mobile telephone companies was notably recognizable, facilitating the USSD service to ensure that the service is not only scalable but also technologically viable to continue to the present [16,33]:

It was suggested [in the meeting] that one should look for risk sharing agreements that create long term possibilities and business models. Look into ways to make MomConnect more sustainable and generate funds.

### Findings Emanating From MomConnect Task Team Interviews

Whereas the previous section focused on analyzing data from indirect engagement with the participants (MomConnect Task Team members), the current section premises on directly obtained data with the self-same participant category through the individual interviews. These themes are discussed in the next sections.

#### Support for MomConnect as a Case Example

Support for an innovative digital health strategy by relevant stakeholders is critical for its long-term sustainability [2,5]. In the case of MomConnect, there was significant support at the different levels of the implementation value chain:

It’s important, if eHealth has to be implemented there has to be somebody with clinical background, who understand the technicalities required to support...Everybody realized this was NDOH project and were keen to be involved.

#### Differing Piloting Views

It is expected that MomConnect content should be in line with, and not deviate from, maternal health guidelines as a contribution to advancing the goals of the country’s maternal health program. However, participants had different views on the use and application of MomConnect as an incipient digital health project of the NDOH through its digital HIS strategy. Concerns were raised about the likelihood of a disjuncture between strategy or policy development and implementation:

I have worked with both eHealth and mHealth strategies outside MomConnect in my previous role...My general assessment about those documents [guidelines] is that they are very theoretical and difficult to implement in real terms, especially the eHealth strategy...[it] is very high-level and doesn’t really explain concretely how to do the things that are recommended.

Although it emphasizes the perspective of incongruences, participants raised concerns about the political overtones of the environment within which the DHIE exists. Such a politicized context could be detrimental to the sustainability of the MomConnect project when politicians subsume the roles of experts, implementers, and other practitioners [14].

Possible reasons for the different responses were that some participants were involved in the drafting of the strategy because they have been working with the Department on different eHealth initiatives and knew what the strategy entails. There were also participants who felt that their roles were very operational and did not need any reference to the strategy. There was also a response that an international organization visited the MomConnect office to benchmark the initiative, and it was difficult to explain the relationship between the initiative and the strategy because the particular organization expected a clear link between the strategy and the initiative. The participants further intimated that there was no indication that MomConnect was adequately responsive to a viable eHealth or mHealth strategy. It was perceived more as a high-level initiative of the Department and replete with policy *speak* with no significant evidence of a sustained national rollout other than addressing the political egos of those connected to the highest echelons of power in the Department:

I have worked with both eHealth and mHealth strategies outside MomConnect in my previous role...My general assessment about those documents [guidelines] is that they are very theoretical and difficult to implement in real terms, especially the eHealth strategy...[it] is very high-level and doesn’t really explain concretely how to do the things that are recommended.

#### Critical Considerations

It emerged during the interviews that the MomConnect Task Team critically considered a range of issues, most prominently the life span of MomConnect within NDOH (its integration within health programming), ethical service implementation, and uncertainty over its sustainability. There were views that this initiative was not integrated with other programs within the Department and that it was implemented as a solo high-profile initiative of the former health minister. The issues around integration included, but were not limited to, data, compliance with HIS reporting, and data management. For instance, the HIS unit has specific rules, but they were perceived to be not fully applied by the MomConnect team:

It has to be integrated within the provincial maternal services. MomConnect is seen as separate entry and is not part of, look at the two units, HIS and HIV/AIDS.

...one of the key issues with any strategy in the space of health technology...is transforming...we have a health system that is changing...having a strategy of five years is actually too little...

#### Ministry of Health Prerogatives

The Ministry of Health is the de jure custodian of the MomConnect digital system, and as such, certain fiduciary responsibilities and expectations are executed through the NDOH [6]. Chiefly, these include leadership, change and stakeholder management, operations, and user-centered design. Collectively, these prerogatives are indicative of attempts to ensure sustained longevity of the overall health digital innovation strategy of the Department [5].

#### Leadership and Change and Stakeholder Management

Some concerns were raised regarding the management of stakeholders:

To have leadership from the NDoH is key. MomConnect has been good at leadership coming from the department. Nobody does anything unless the NDoH signed on it.

On change management, the following excerpt captures the unanimous views of the interviewees in this regard:

There are no contracts. All partners are taking some level of risk, no formal agreement to say “this is how we will sustain the programme going forward. We need proper project management and change management strategy.”

As opposed to the policy-level sites of the MACeH and MomConnect Task Team, the health care facilities (especially) at the local and district levels are at the coalface of the MomConnect system’s implementation [6,33].

#### Clinical Staff (Nurses)

Staff expressed that they felt excluded and alienated, as they were not part of MomConnect development. They were only told to register and subscribe patients to it, which was a nonclinical (auxiliary health) function. Nurses reported that the content of the SMS text messages received by the women was never shared with them. They only heard from patients when they were asked for clarity about specific messages or when they confirmed specific elements of ANC. Some nurses even reported subscribing to MomConnect for the purpose of familiarizing themselves with the contents of MomConnect on the end users’ mobile phones. Because nurses do not usually register patients on MomConnect, the counselors and health promoters complained about the additional workload caused by the time taken to register users on the MomConnect platform:

I only heard of MomConnect 4 weeks ago when I started in antenatal care. I do not know the content of the messages our patients receive. We need to get the messages so that the content can be used as part of health education.

...our biggest challenge is language barrier as most of our clients are from out of our South African borders...due to language barriers, patients don’t receive information...

#### Mothers and Pregnant Women

Disgruntlement was expressed by the pregnant women regarding the technicality and language barriers posed by the MomConnect functionality on their mobile devices. The disgruntlement emanated largely from foreign nationals, whose home languages were not part of South Africa’s 11 official languages. As such, they (foreign nationals) frequently experienced communication barriers and understanding of MomConnect content. All pregnant women (regardless of country of origin) also experienced technical problems due to connectivity and time-out sessions induced by USSD operational failures in the provider network system. Notwithstanding, virtually all participating pregnant women expressed their satisfaction with the MomConnect SMS text messages and commended the service on the content regarding nutrition and preparing for the labor trip, and the nontechnical language used was easy to understand. However, there was a preference for the use of English as opposed to their own vernaculars because the translation in their vernacular was perceived as bombastic.

#### Service Implementation

The implementation and national rollout of a digital health service the size of MomConnect is largely dependent on the broad involvement and capacity of stakeholders at the health care facilities as the points of service [9,10]. Accordingly, the (clinical and nonclinical) operational context or environment of the health care facility and its units also determine the level at which the service is offered, whether it is acceptable or satisfactory or otherwise [2,8]. The findings indicated a general level of acceptable service implementation.

## Discussion

### Principal Findings

The principal findings in this study collectively reflect the interconnectedness between the adopted research approach (Table 1), the varied participant categories (Table 2), and the framework within which these findings were formulated (Table 3). The practitioner-researcher approach facilitated the seamlessness of the thematic, discourse or conversational, content, and convergence analytical modes. By virtue of his knowledge and experience as a former MomConnect Task Team member and based on the period during which the study was conducted, the findings are then the product of “...research in practice, a close study of practices with access to pertinent data” [21].

The study’s most fundamental purpose was to use a stakeholder perspective as a preferred method to examine the scalability and sustainability factors of the NDOH’s current MomConnect initiative, from which policy guidelines could be developed to inform on possible areas or aspects requiring improvements. A close observation of the results framework clearly indicates that the findings are located within 2 principal aspects of an innovative digital health ecosystem, namely, the digital environmental factors and the practice-related implementation attributes.

Regarding legislative and policy compliance, there are laws in South Africa, but the implementation of mHealth (and other spheres of public life) has not been consistently enforced. This study did not specifically examine law and health information. However, from the data generated in this study, there is an applicable regulation to mHealth, and implementers are aware of its applicability on mHealth. From the themes, particularly from the facility-level implementers as the service point workforce, a curriculum for eHealth was proposed. However, there is no reported process for the proposed curriculum to be developed or under review.

From a clinical perspective, the effectiveness of mHealth interoperability relies on a partnership between patients and clinicians’ workflows. In particular, mHealth devices would benefit from interoperability standards to ease integration with other health software apps, which are increasingly required to organize the large amounts of data collected [12,15]. In the context of this study, the digital HISs served as the context for MomConnect standards and interoperability. Information regarding full implementation following industry standards and usability across various devices and platforms showed little coverage and application.

Network infrastructure development and readiness were largely facilitated through partnerships with established mobile technology operators, as the NDOH does not have its own. This is an instance of the worth and usefulness of private-public partnerships, especially in a sphere of public life, where technological developments necessitate capital cost sharing (Pillay Y, BP, unpublished data, April 2022) [12]. For network services and device applications, the study confined itself more to the end-user features than to the system design aspects. South Africa’s list of network facilities includes, but is not limited to, geographic information system mapping.

The objective of this study was to review the NDOH’s MomConnect initiative as a case study of an existing government mHealth strategy, followed by the design of an improved strategy based on best demonstrated practices (considerations and methods) and learned experiences from the perspectives of multiple stakeholders within the DHIE in South Africa.

### Strengths and Limitations

This study is strengthened by its user- and stakeholder-focused orientation, which accommodated both the nonprobability judgment and convenience sampling strategies. Many studies in this field are more technology or device-oriented and focus on *high-end* users of mobile technology. In contrast, the study could have a limited reach insofar as its confinement to PHC users in a metropolitan inner-city area, which might have different comparable outcomes in rural, suburban, and condensed informal settlement contexts. However, a significant aspect of the broader limitations was mitigated by the heterogeneity of the sampled participant categories representing different *vested* interests in society.

### Future Research Directions

Further research is needed for the retrospective analysis of mHealth initiatives and the forecasting of the sustainability of current and future mHealth initiatives in South Africa, that is, the cumulative effect and impact of mHealth strategies and projects with similar or more emphasis accorded by the NDOH. In addition, more research is required from public health practitioners in practice on mHealth, as they were in daily contact with the mHealth beneficiaries (end users), which aptly placed them to obtain first-hand stakeholder perspectives and experiences [9,20].

### Conclusions

The fact that MomConnect is generally a usable technology-based communication system does not preclude the identification of alternative interventions to ameliorate or even radically improve its shortcomings. For instance, the current caveat emptor approach renders pregnant women vulnerable to the same challenges generally experienced by most South Africans with internet-based broadband distribution. Notwithstanding the information distribution efficiency for antenatal and postnatal purposes, the USSD mechanism still allocates a cost to the user, albeit less than the sometimes prohibitive costs of data compared with the rest of the world. Therefore, it is unsurprising that the main critical considerations were funding for eHealth, including mHealth. Sustainability is a concept that must be considered throughout the implementation process of the MomConnect project. However, there was less consideration at the beginning of this initiative because we could consider sustaining something that has not even scaled. Funding for eHealth, including mHealth, is critical. The government must have its own funding mechanisms and must not depend only on funders.

## Abbreviations

ANC: antenatal care
DHIE: digital health innovation ecosystem
HIS: health information system
ICT: information and communication technology
MACeH: Ministerial Advisory Committee on eHealth
MCWH: maternal, child, and women’s health
mHealth: mobile health
NDHS: National Digital Health Strategy
NDOH: National Department of Health
PHC: primary health care
USSD: Unstructured Supplementary Service Data
